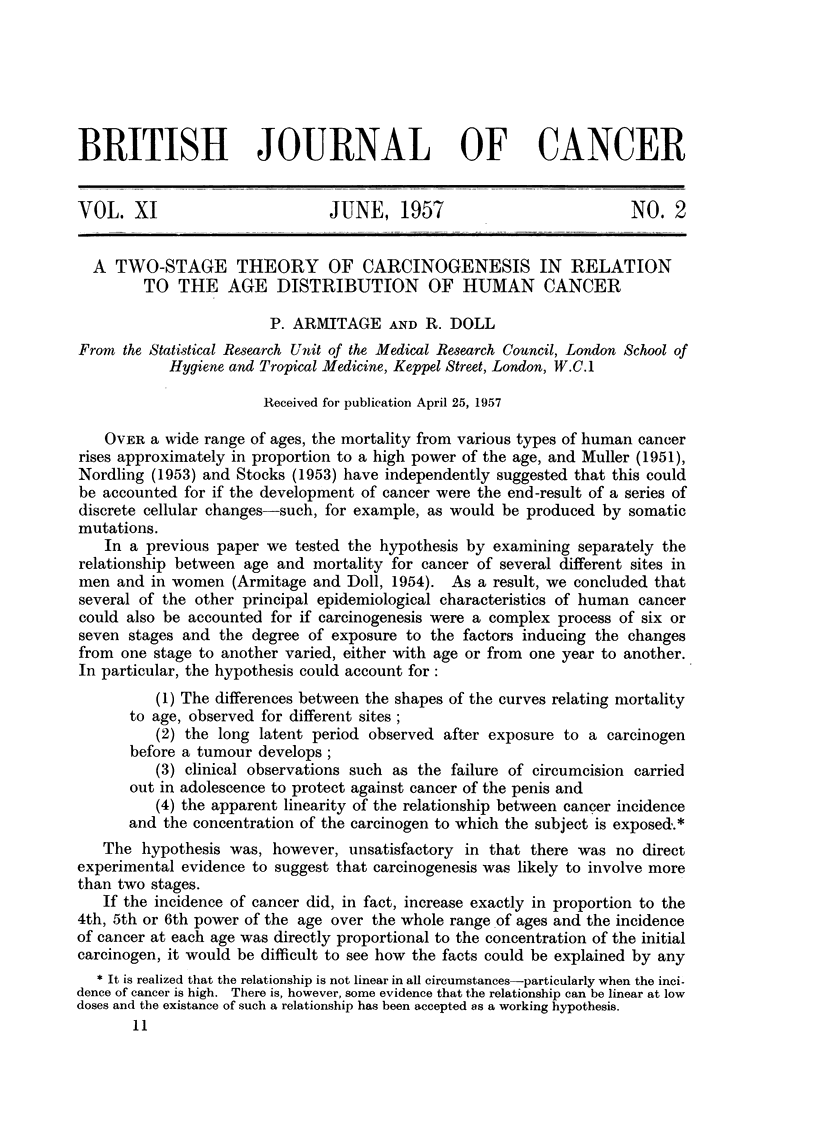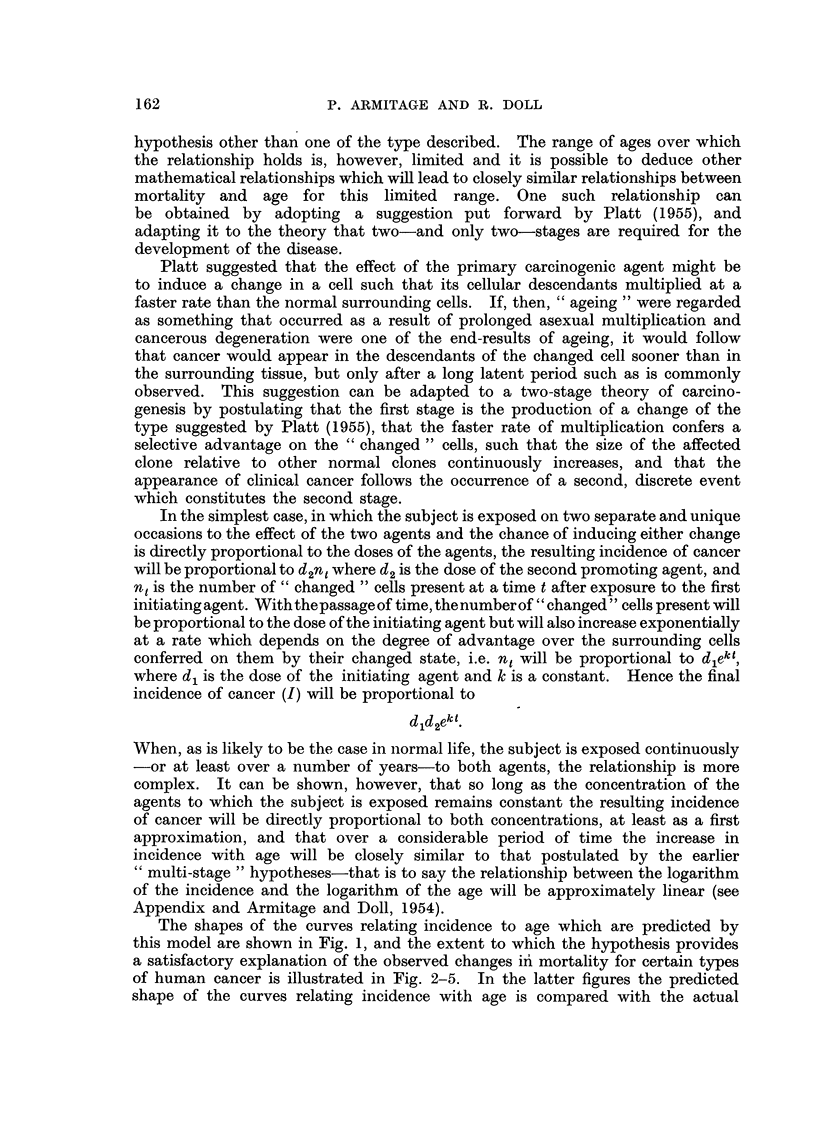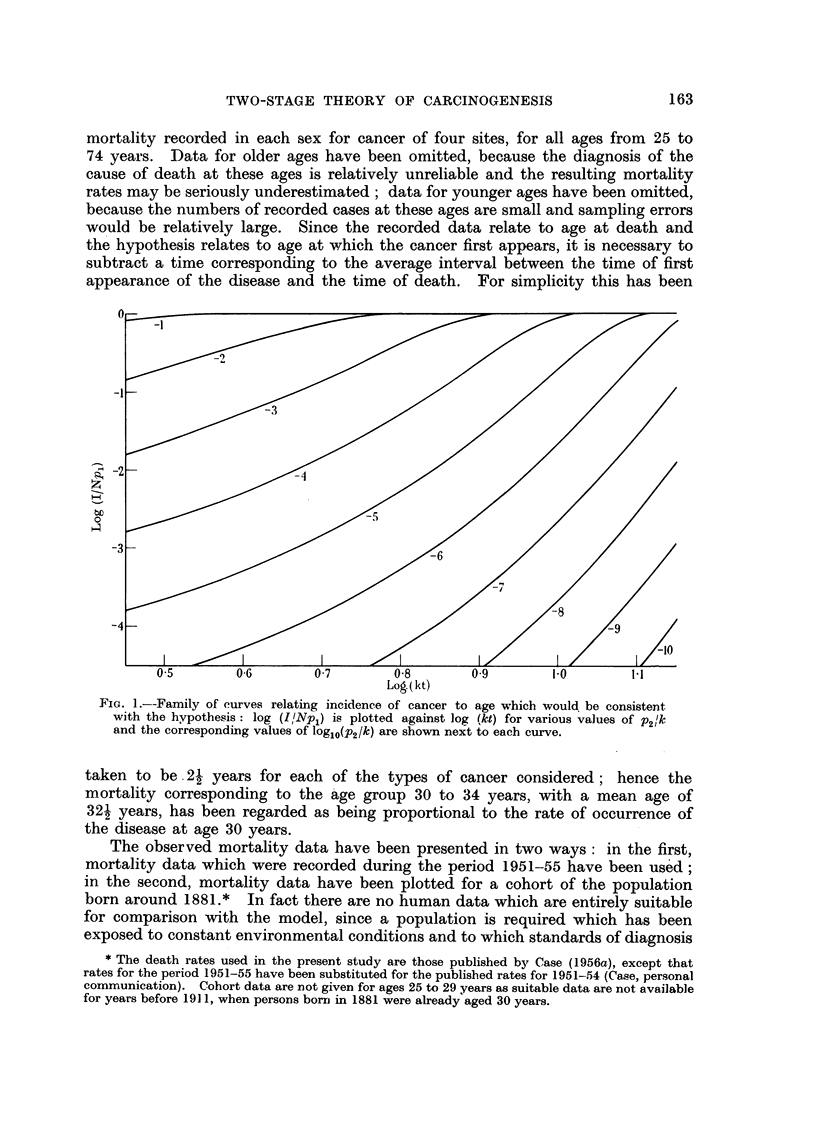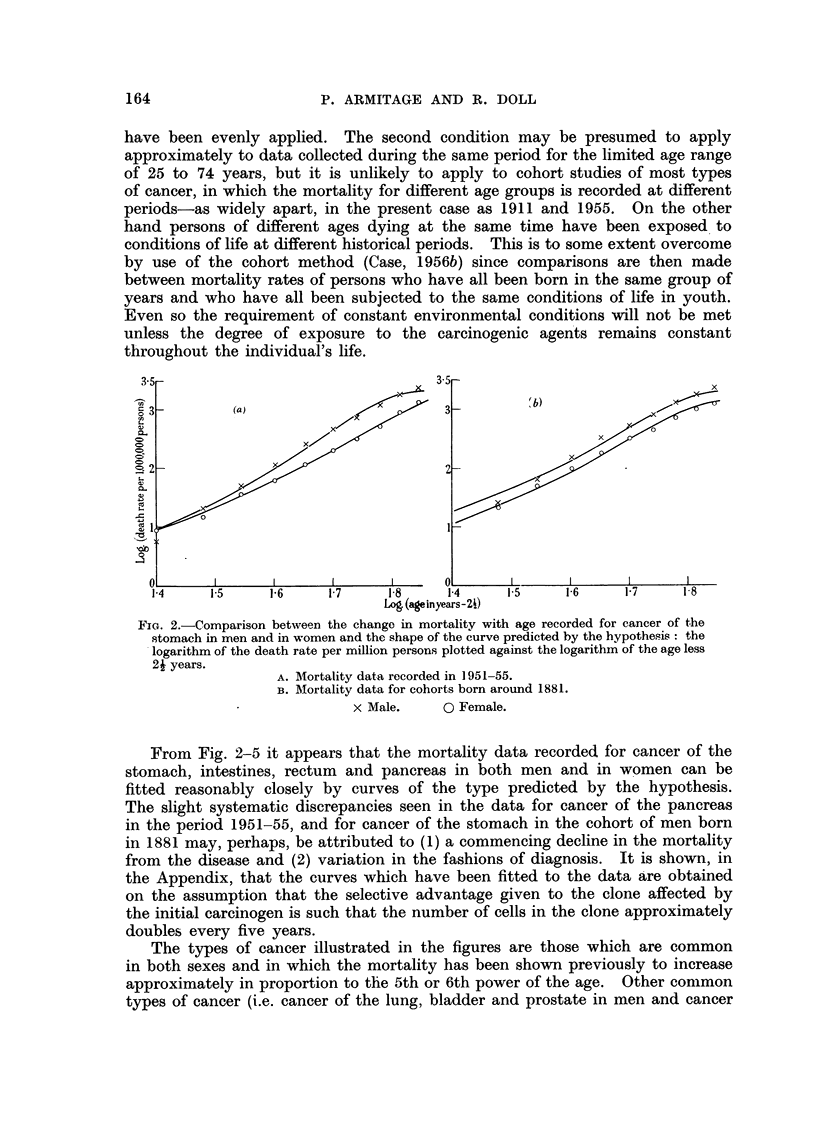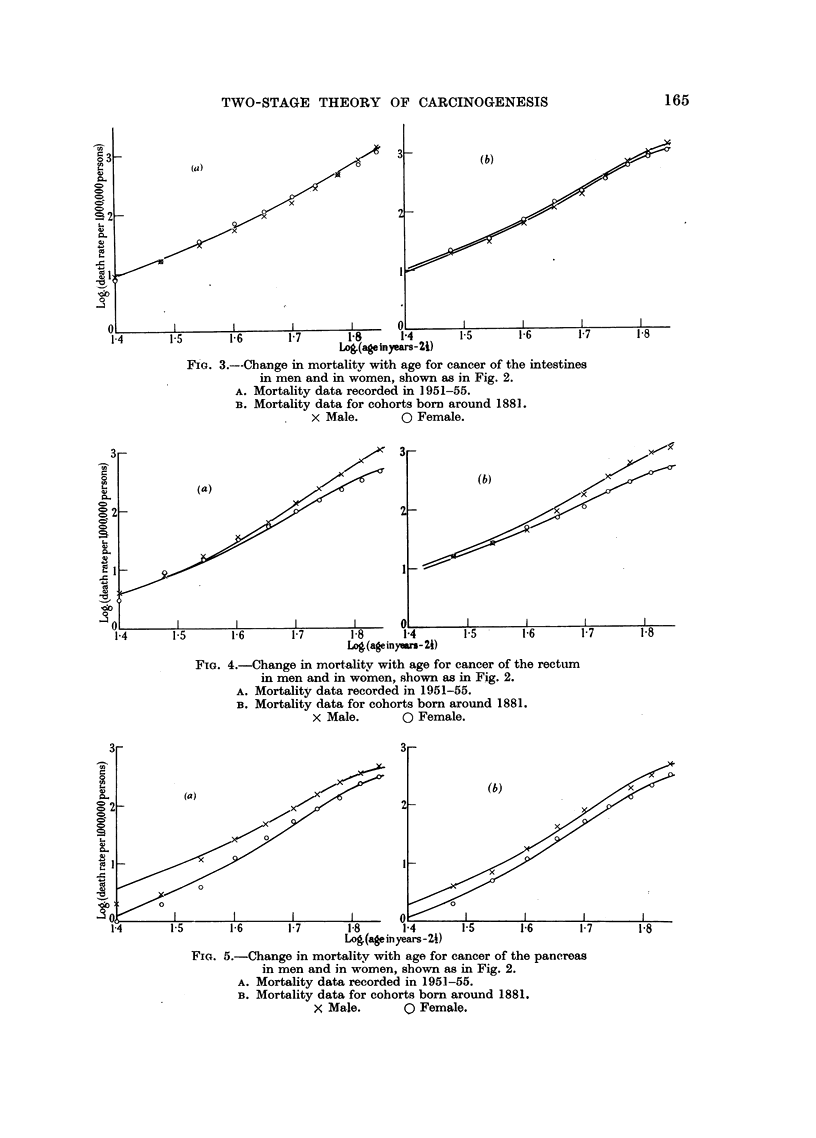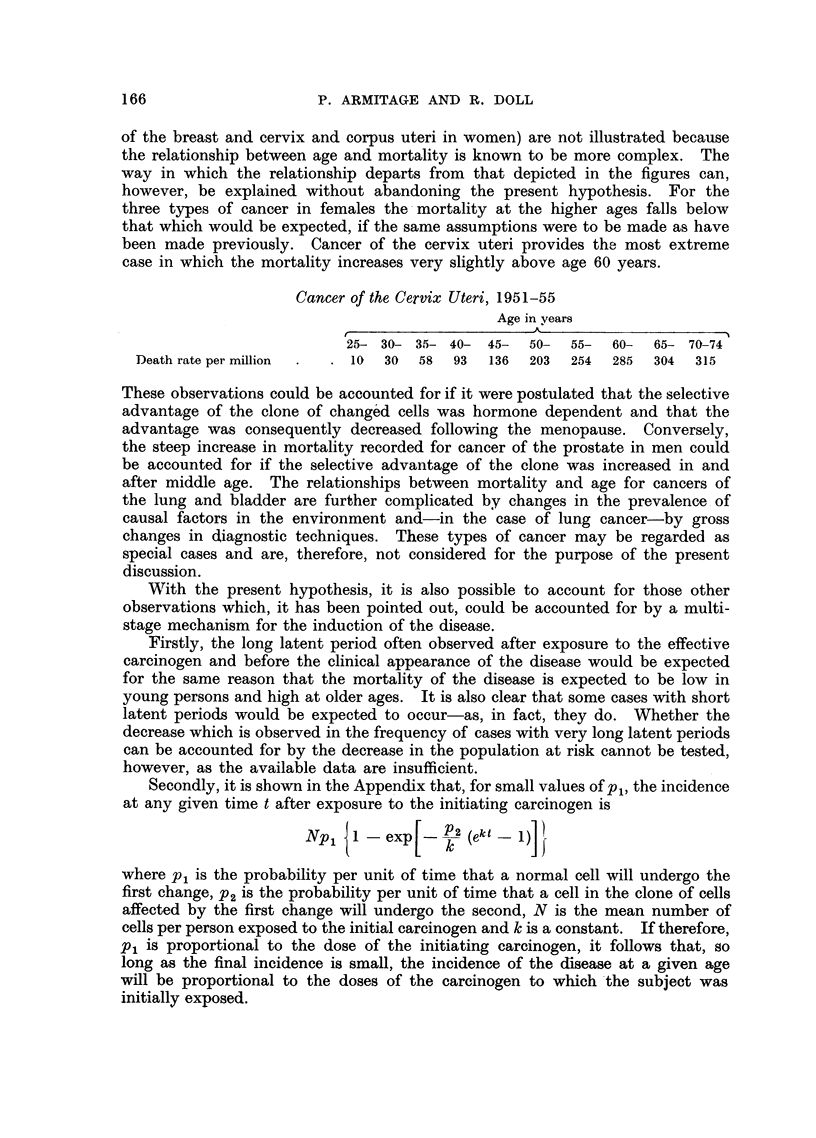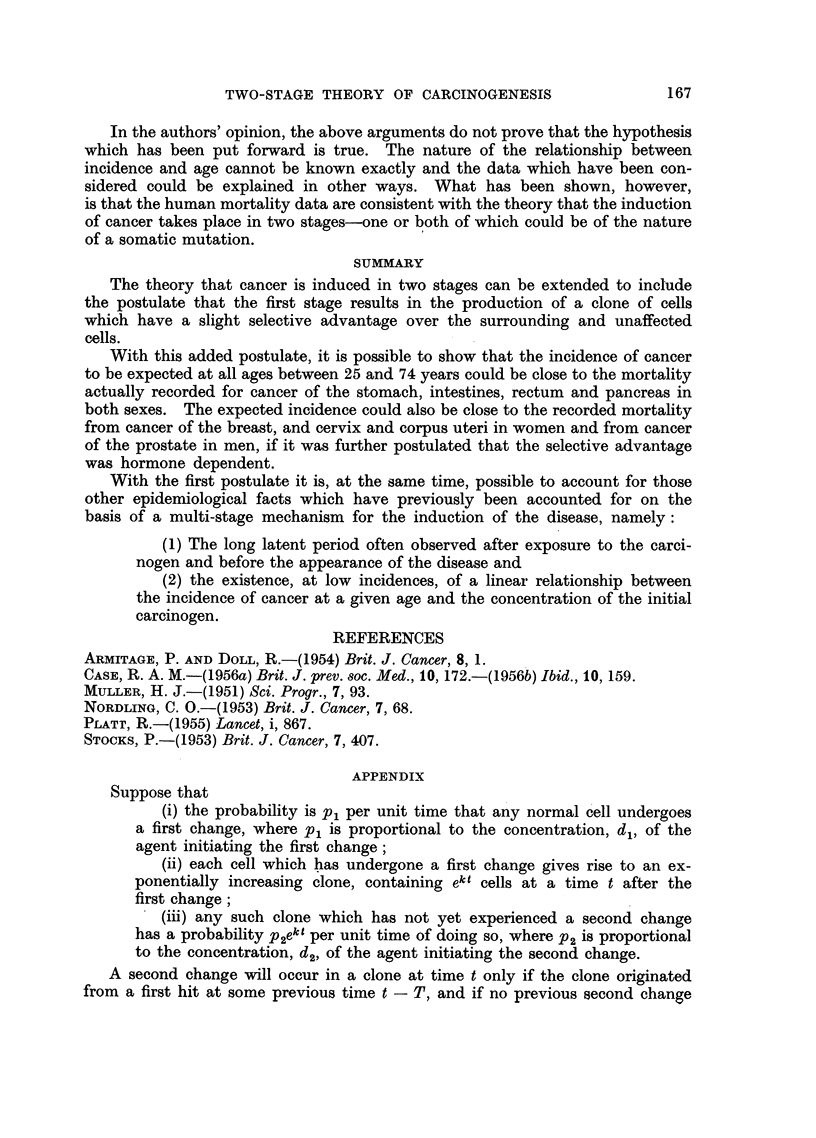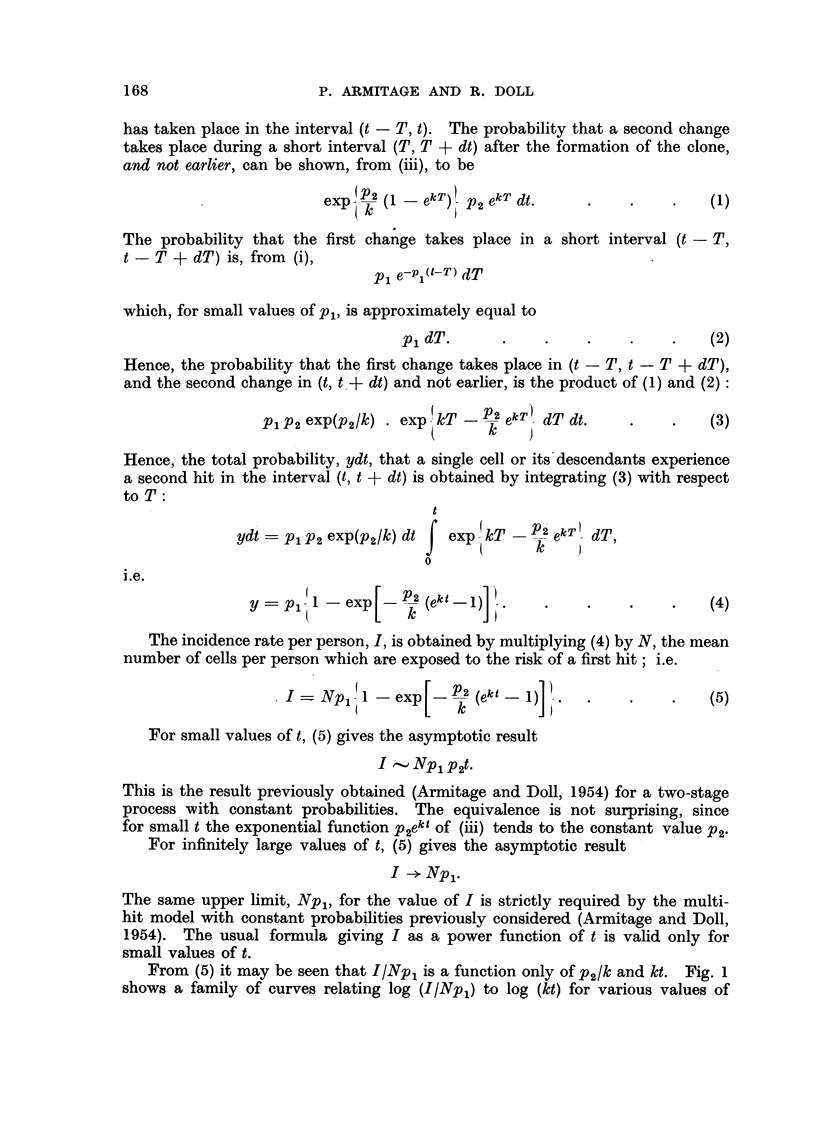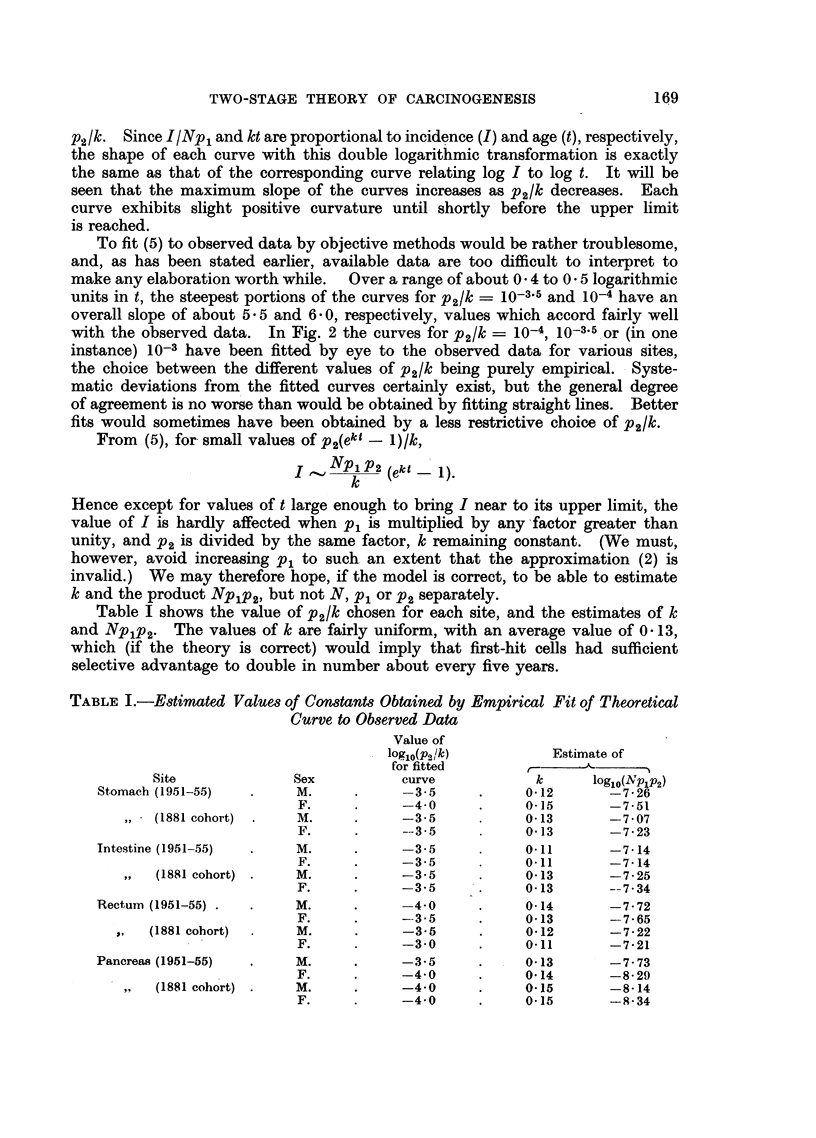# A Two-stage Theory of Carcinogenesis in Relation to the Age Distribution of Human Cancer

**DOI:** 10.1038/bjc.1957.22

**Published:** 1957-06

**Authors:** P. Armitage, R. Doll


BRITISH JOURNAL OF CANCER

VOL. XI JUNE, 1957 NO. 2

### A TWO-STAGE THEORY OF CARCINOGENESIS IN RELATION TO THE AGE DISTRIBUTION OF HUMAN CANCER

P. ARMITAGE AND R. DOLL

*From the Statistical Research Unit of the Medical Research Council, London School of Hygiene and Tropical Medicine, Keppel Street, London, W.C.*1

Received for publication April 25, 1957

OVER a wide range of ages, the mortality from various types of human cancer rises approximately in proportion to a high power of the age, and Muller (1951), Nordling (1953) and Stocks (1953) have independently suggested that this could be accounted for if the development of cancer were the end-result of a series of discrete cellular changes—such, for example, as would be produced by somatic mutations.

In a previous paper we tested the hypothesis by examining separately the relationship between age and mortality for cancer of several different sites in men and in women (Armitage and Doll, 1954). As a result, we concluded that several of the other principal epidemiological characteristics of human cancer could also be accounted for if carcinogenesis were a complex process of six or seven stages and the degree of exposure to the factors inducing the changes from one stage to another varied, either with age or from one year to another. In particular, the hypothesis could account for :

(1) The differences between the shapes of the curves relating mortality to age, observed for different sites ;
(2) the long latent period observed after exposure to a carcinogen before a tumour develops ;
(3) clinical observations such as the failure of circumcision carried out in adolescence to protect against cancer of the penis and
(4) the apparent linearity of the relationship between cancer incidence and the concentration of the carcinogen to which the subject is exposed.*

The hypothesis was, however, unsatisfactory in that there was no direct experimental evidence to suggest that carcinogenesis was likely to involve more than two stages.

If the incidence of cancer did, in fact, increase exactly in proportion to the 4th, 5th or 6th power of the age over the whole range of ages and the incidence of cancer at each age was directly proportional to the concentration of the initial carcinogen, it would be difficult to see how the facts could be explained by any

* It is realized that the relationship is not linear in all circumstances—particularly when the incidence of cancer is high. There is, however, some evidence that the relationship can be linear at low doses and the existance of such a relationship has been accepted as a working hypothesis.

hypothesis other than one of the type described. The range of ages over which the relationship holds is, however, limited and it is possible to deduce other mathematical relationships which will lead to closely similar relationships between mortality and age for this limited range. One such relationship can be obtained by adopting a suggestion put forward by Platt (1955), and adapting it to the theory that two—and only two—stages are required for the development of the disease.

Platt suggested that the effect of the primary carcinogenic agent might be to induce a change in a cell such that its cellular descendants multiplied at a faster rate than the normal surrounding cells. If, then, " ageing " were regarded as something that occurred as a result of prolonged asexual multiplication and cancerous degeneration were one of the end-results of ageing, it would follow that cancer would appear in the descendants of the changed cell sooner than in the surrounding tissue, but only after a long latent period such as is commonly observed. This suggestion can be adapted to a two-stage theory of carcinogenesis by postulating that the first stage is the production of a change of the type suggested by Platt (1955), that the faster rate of multiplication confers a selective advantage on the " changed " cells, such that the size of the affected clone relative to other normal clones continuously increases, and that the appearance of clinical cancer follows the occurrence of a second, discrete event which constitutes the second stage.

In the simplest case, in which the subject is exposed on two separate and unique occasions to the effect of the two agents and the chance of inducing either change is directly proportional to the doses of the agents, the resulting incidence of cancer will be proportional to $d_2 n_t$ where $d_2$ is the dose of the second promoting agent, and $n_t$ is the number of " changed " cells present at a time $t$ after exposure to the first initiating agent. With the passage of time, the number of "changed" cells present will be proportional to the dose of the initiating agent but will also increase exponentially at a rate which depends on the degree of advantage over the surrounding cells conferred on them by their changed state, i.e. $n_t$ will be proportional to $d_1 e^{kt}$, where $d_1$ is the dose of the initiating agent and $k$ is a constant. Hence the final incidence of cancer ($I$) will be proportional to

$$d_1 d_2 e^{kt}.$$

When, as is likely to be the case in normal life, the subject is exposed continuously —or at least over a number of years—to both agents, the relationship is more complex. It can be shown, however, that so long as the concentration of the agents to which the subject is exposed remains constant the resulting incidence of cancer will be directly proportional to both concentrations, at least as a first approximation, and that over a considerable period of time the increase in incidence with age will be closely similar to that postulated by the earlier " multi-stage " hypotheses—that is to say the relationship between the logarithm of the incidence and the logarithm of the age will be approximately linear (see Appendix and Armitage and Doll, 1954).

The shapes of the curves relating incidence to age which are predicted by this model are shown in Fig. 1, and the extent to which the hypothesis provides a satisfactory explanation of the observed changes in mortality for certain types of human cancer is illustrated in Fig. 2–5. In the latter figures the predicted shape of the curves relating incidence with age is compared with the actual mortality recorded in each sex for cancer of four sites, for all ages from 25 to 74 years. Data for older ages have been omitted, because the diagnosis of the cause of death at these ages is relatively unreliable and the resulting mortality rates may be seriously underestimated ; data for younger ages have been omitted, because the numbers of recorded cases at these ages are small and sampling errors would be relatively large. Since the recorded data relate to age at death and the hypothesis relates to age at which the cancer first appears, it is necessary to subtract a time corresponding to the average interval between the time of first appearance of the disease and the time of death. For simplicity this has been

FIG. 1.—Family of curves relating incidence of cancer to age which would be consistent with the hypothesis : log $(I/Np_1)$ is plotted against log $(kt)$ for various values of $p_2/k$ and the corresponding values of $\log_{10}(p_2/k)$ are shown next to each curve.

taken to be $2\frac{1}{2}$ years for each of the types of cancer considered ; hence the mortality corresponding to the age group 30 to 34 years, with a mean age of $32\frac{1}{2}$ years, has been regarded as being proportional to the rate of occurrence of the disease at age 30 years.

The observed mortality data have been presented in two ways : in the first, mortality data which were recorded during the period 1951–55 have been used ; in the second, mortality data have been plotted for a cohort of the population born around 1881.* In fact there are no human data which are entirely suitable for comparison with the model, since a population is required which has been exposed to constant environmental conditions and to which standards of diagnosis

* The death rates used in the present study are those published by Case (1956*a*), except that rates for the period 1951–55 have been substituted for the published rates for 1951–54 (Case, personal communication). Cohort data are not given for ages 25 to 29 years as suitable data are not available for years before 1911, when persons born in 1881 were already aged 30 years.

have been evenly applied. The second condition may be presumed to apply approximately to data collected during the same period for the limited age range of 25 to 74 years, but it is unlikely to apply to cohort studies of most types of cancer, in which the mortality for different age groups is recorded at different periods—as widely apart, in the present case as 1911 and 1955. On the other hand persons of different ages dying at the same time have been exposed to conditions of life at different historical periods. This is to some extent overcome by use of the cohort method (Case, 1956*b*) since comparisons are then made between mortality rates of persons who have all been born in the same group of years and who have all been subjected to the same conditions of life in youth. Even so the requirement of constant environmental conditions will not be met unless the degree of exposure to the carcinogenic agents remains constant throughout the individual's life.

Fig. 2.—Comparison between the change in mortality with age recorded for cancer of the stomach in men and in women and the shape of the curve predicted by the hypothesis : the logarithm of the death rate per million persons plotted against the logarithm of the age less 2½ years.

A. Mortality data recorded in 1951–55.
B. Mortality data for cohorts born around 1881.
× Male. ○ Female.

From Fig. 2–5 it appears that the mortality data recorded for cancer of the stomach, intestines, rectum and pancreas in both men and in women can be fitted reasonably closely by curves of the type predicted by the hypothesis. The slight systematic discrepancies seen in the data for cancer of the pancreas in the period 1951–55, and for cancer of the stomach in the cohort of men born in 1881 may, perhaps, be attributed to (1) a commencing decline in the mortality from the disease and (2) variation in the fashions of diagnosis. It is shown, in the Appendix, that the curves which have been fitted to the data are obtained on the assumption that the selective advantage given to the clone affected by the initial carcinogen is such that the number of cells in the clone approximately doubles every five years.

The types of cancer illustrated in the figures are those which are common in both sexes and in which the mortality has been shown previously to increase approximately in proportion to the 5th or 6th power of the age. Other common types of cancer (i.e. cancer of the lung, bladder and prostate in men and cancer

FIG. 3.—Change in mortality with age for cancer of the intestines in men and in women, shown as in Fig. 2.
A. Mortality data recorded in 1951–55.
B. Mortality data for cohorts born around 1881.
× Male. ○ Female.

FIG. 4.—Change in mortality with age for cancer of the rectum in men and in women, shown as in Fig. 2.
A. Mortality data recorded in 1951–55.
B. Mortality data for cohorts born around 1881.
× Male. ○ Female.

FIG. 5.—Change in mortality with age for cancer of the pancreas in men and in women, shown as in Fig. 2.
A. Mortality data recorded in 1951–55.
B. Mortality data for cohorts born around 1881.
× Male. ○ Female.

of the breast and cervix and corpus uteri in women) are not illustrated because the relationship between age and mortality is known to be more complex. The way in which the relationship departs from that depicted in the figures can, however, be explained without abandoning the present hypothesis. For the three types of cancer in females the mortality at the higher ages falls below that which would be expected, if the same assumptions were to be made as have been made previously. Cancer of the cervix uteri provides the most extreme case in which the mortality increases very slightly above age 60 years.

*Cancer of the Cervix Uteri*, 1951–55

| | Age in years | | | | | | | | | |
|---|---|---|---|---|---|---|---|---|---|---|
| | 25– | 30– | 35– | 40– | 45– | 50– | 55– | 60– | 65– | 70–74 |
| Death rate per million | 10 | 30 | 58 | 93 | 136 | 203 | 254 | 285 | 304 | 315 |

These observations could be accounted for if it were postulated that the selective advantage of the clone of changed cells was hormone dependent and that the advantage was consequently decreased following the menopause. Conversely, the steep increase in mortality recorded for cancer of the prostate in men could be accounted for if the selective advantage of the clone was increased in and after middle age. The relationships between mortality and age for cancers of the lung and bladder are further complicated by changes in the prevalence of causal factors in the environment and—in the case of lung cancer—by gross changes in diagnostic techniques. These types of cancer may be regarded as special cases and are, therefore, not considered for the purpose of the present discussion.

With the present hypothesis, it is also possible to account for those other observations which, it has been pointed out, could be accounted for by a multistage mechanism for the induction of the disease.

Firstly, the long latent period often observed after exposure to the effective carcinogen and before the clinical appearance of the disease would be expected for the same reason that the mortality of the disease is expected to be low in young persons and high at older ages. It is also clear that some cases with short latent periods would be expected to occur—as, in fact, they do. Whether the decrease which is observed in the frequency of cases with very long latent periods can be accounted for by the decrease in the population at risk cannot be tested, however, as the available data are insufficient.

Secondly, it is shown in the Appendix that, for small values of $p_1$, the incidence at any given time $t$ after exposure to the initiating carcinogen is

$$Np_1 \left\{1 - \exp\left[-\frac{p_2}{k}(e^{kt} - 1)\right]\right\}$$

where $p_1$ is the probability per unit of time that a normal cell will undergo the first change, $p_2$ is the probability per unit of time that a cell in the clone of cells affected by the first change will undergo the second, $N$ is the mean number of cells per person exposed to the initial carcinogen and $k$ is a constant. If therefore, $p_1$ is proportional to the dose of the initiating carcinogen, it follows that, so long as the final incidence is small, the incidence of the disease at a given age will be proportional to the doses of the carcinogen to which the subject was initially exposed.

In the authors' opinion, the above arguments do not prove that the hypothesis which has been put forward is true. The nature of the relationship between incidence and age cannot be known exactly and the data which have been considered could be explained in other ways. What has been shown, however, is that the human mortality data are consistent with the theory that the induction of cancer takes place in two stages—one or both of which could be of the nature of a somatic mutation.

### SUMMARY

The theory that cancer is induced in two stages can be extended to include the postulate that the first stage results in the production of a clone of cells which have a slight selective advantage over the surrounding and unaffected cells.

With this added postulate, it is possible to show that the incidence of cancer to be expected at all ages between 25 and 74 years could be close to the mortality actually recorded for cancer of the stomach, intestines, rectum and pancreas in both sexes. The expected incidence could also be close to the recorded mortality from cancer of the breast, and cervix and corpus uteri in women and from cancer of the prostate in men, if it was further postulated that the selective advantage was hormone dependent.

With the first postulate it is, at the same time, possible to account for those other epidemiological facts which have previously been accounted for on the basis of a multi-stage mechanism for the induction of the disease, namely:

(1) The long latent period often observed after exposure to the carcinogen and before the appearance of the disease and

(2) the existence, at low incidences, of a linear relationship between the incidence of cancer at a given age and the concentration of the initial carcinogen.

### REFERENCES

Armitage, P. and Doll, R.—(1954) *Brit. J. Cancer*, **8**, 1.
Case, R. A. M.—(1956*a*) *Brit. J. prev. soc. Med.*, **10**, 172.—(1956*b*) *Ibid.*, **10**, 159.
Muller, H. J.—(1951) *Sci. Progr.*, **7**, 93.
Nordling, C. O.—(1953) *Brit. J. Cancer*, **7**, 68.
Platt, R.—(1955) *Lancet*, i, 867.
Stocks, P.—(1953) *Brit. J. Cancer*, **7**, 407.

### APPENDIX

Suppose that

(i) the probability is $p_1$ per unit time that any normal cell undergoes a first change, where $p_1$ is proportional to the concentration, $d_1$, of the agent initiating the first change;

(ii) each cell which has undergone a first change gives rise to an exponentially increasing clone, containing $e^{kt}$ cells at a time $t$ after the first change;

(iii) any such clone which has not yet experienced a second change has a probability $p_2e^{kt}$ per unit time of doing so, where $p_2$ is proportional to the concentration, $d_2$, of the agent initiating the second change.

A second change will occur in a clone at time $t$ only if the clone originated from a first hit at some previous time $t - T$, and if no previous second change has taken place in the interval $(t - T, t)$. The probability that a second change takes place during a short interval $(T, T + dt)$ after the formation of the clone, *and not earlier*, can be shown, from (iii), to be

$$\exp\left\{\frac{p_2}{k}(1 - e^{kT})\right\} p_2 e^{kT} dt. \quad . \quad . \quad . \quad . \quad (1)$$

The probability that the first change takes place in a short interval $(t - T, t - T + dT)$ is, from (i),

$$p_1 e^{-p_1(t-T)} dT$$

which, for small values of $p_1$, is approximately equal to

$$p_1 dT. \quad . \quad . \quad . \quad . \quad . \quad . \quad (2)$$

Hence, the probability that the first change takes place in $(t - T, t - T + dT)$, and the second change in $(t, t + dt)$ and not earlier, is the product of (1) and (2) :

$$p_1 p_2 \exp(p_2/k) \, . \, \exp\left\{kT - \frac{p_2}{k} e^{kT}\right\} dT\, dt. \quad . \quad . \quad (3)$$

Hence, the total probability, $ydt$, that a single cell or its descendants experience a second hit in the interval $(t, t + dt)$ is obtained by integrating (3) with respect to $T$ :

$$ydt = p_1 p_2 \exp(p_2/k)\, dt \int_0^t \exp\left\{kT - \frac{p_2}{k} e^{kT}\right\} dT,$$

i.e.

$$y = p_1\left\{1 - \exp\left[-\frac{p_2}{k}(e^{kt} - 1)\right]\right\}. \quad . \quad . \quad . \quad . \quad . \quad (4)$$

The incidence rate per person, $I$, is obtained by multiplying (4) by $N$, the mean number of cells per person which are exposed to the risk of a first hit ; i.e.

$$I = Np_1\left\{1 - \exp\left[-\frac{p_2}{k}(e^{kt} - 1)\right]\right\}. \quad . \quad . \quad . \quad . \quad (5)$$

For small values of $t$, (5) gives the asymptotic result

$$I \sim Np_1 p_2 t.$$

This is the result previously obtained (Armitage and Doll, 1954) for a two-stage process with constant probabilities. The equivalence is not surprising, since for small $t$ the exponential function $p_2 e^{kt}$ of (iii) tends to the constant value $p_2$.

For infinitely large values of $t$, (5) gives the asymptotic result

$$I \to Np_1.$$

The same upper limit, $Np_1$, for the value of $I$ is strictly required by the multi-hit model with constant probabilities previously considered (Armitage and Doll, 1954). The usual formula giving $I$ as a power function of $t$ is valid only for small values of $t$.

From (5) it may be seen that $I/Np_1$ is a function only of $p_2/k$ and $kt$. Fig. 1 shows a family of curves relating log $(I/Np_1)$ to log $(kt)$ for various values of

$p_2/k$. Since $I/Np_1$ and $kt$ are proportional to incidence ($I$) and age ($t$), respectively, the shape of each curve with this double logarithmic transformation is exactly the same as that of the corresponding curve relating log $I$ to log $t$. It will be seen that the maximum slope of the curves increases as $p_2/k$ decreases. Each curve exhibits slight positive curvature until shortly before the upper limit is reached.

To fit (5) to observed data by objective methods would be rather troublesome, and, as has been stated earlier, available data are too difficult to interpret to make any elaboration worth while. Over a range of about 0·4 to 0·5 logarithmic units in $t$, the steepest portions of the curves for $p_2/k = 10^{-3\cdot5}$ and $10^{-4}$ have an overall slope of about 5·5 and 6·0, respectively, values which accord fairly well with the observed data. In Fig. 2 the curves for $p_2/k = 10^{-4}$, $10^{-3\cdot5}$ or (in one instance) $10^{-3}$ have been fitted by eye to the observed data for various sites, the choice between the different values of $p_2/k$ being purely empirical. Systematic deviations from the fitted curves certainly exist, but the general degree of agreement is no worse than would be obtained by fitting straight lines. Better fits would sometimes have been obtained by a less restrictive choice of $p_2/k$.

From (5), for small values of $p_2(e^{kt} - 1)/k$,

$$I \sim \frac{Np_1 p_2}{k} (e^{kt} - 1).$$

Hence except for values of $t$ large enough to bring $I$ near to its upper limit, the value of $I$ is hardly affected when $p_1$ is multiplied by any factor greater than unity, and $p_2$ is divided by the same factor, $k$ remaining constant. (We must, however, avoid increasing $p_1$ to such an extent that the approximation (2) is invalid.) We may therefore hope, if the model is correct, to be able to estimate $k$ and the product $Np_1p_2$, but not $N$, $p_1$ or $p_2$ separately.

Table I shows the value of $p_2/k$ chosen for each site, and the estimates of $k$ and $Np_1p_2$. The values of $k$ are fairly uniform, with an average value of 0·13, which (if the theory is correct) would imply that first-hit cells had sufficient selective advantage to double in number about every five years.

TABLE I.—*Estimated Values of Constants Obtained by Empirical Fit of Theoretical Curve to Observed Data*

| Site | Sex | Value of $\log_{10}(p_2/k)$ for fitted curve | Estimate of $k$ | Estimate of $\log_{10}(Np_1p_2)$ |
|---|---|---|---|---|
| Stomach (1951–55) | M. | −3·5 | 0·12 | −7·26 |
| | F. | −4·0 | 0·15 | −7·51 |
| ,, (1881 cohort) | M. | −3·5 | 0·13 | −7·07 |
| | F. | −3·5 | 0·13 | −7·23 |
| Intestine (1951–55) | M. | −3·5 | 0·11 | −7·14 |
| | F. | −3·5 | 0·11 | −7·14 |
| ,, (1881 cohort) | M. | −3·5 | 0·13 | −7·25 |
| | F. | −3·5 | 0·13 | −7·34 |
| Rectum (1951–55) | M. | −4·0 | 0·14 | −7·72 |
| | F. | −3·5 | 0·13 | −7·65 |
| ,, (1881 cohort) | M. | −3·5 | 0·12 | −7·22 |
| | F. | −3·0 | 0·11 | −7·21 |
| Pancreas (1951–55) | M. | −3·5 | 0·13 | −7·73 |
| | F. | −4·0 | 0·14 | −8·29 |
| ,, (1881 cohort) | M. | −4·0 | 0·15 | −8·14 |
| | F. | −4·0 | 0·15 | −8·34 |